# Monitoring the variations in physicochemical characteristics of squab meat during the braising cooking process

**DOI:** 10.1002/fsn3.2876

**Published:** 2022-04-07

**Authors:** Wenhong Zhao, Qiaoyu Liu, Hao Jiang, Minyi Zheng, Min Qian, Xiaofang Zeng, Weidong Bai

**Affiliations:** ^1^ Guangdong Provincial Key Laboratory of Lingnan Specialty Food Science and Technology Zhongkai University of Agriculture and Engineering Guangzhou 510225 China; ^2^ College of Light Industry and Food Sciences Zhongkai University of Agriculture and Engineering Guangzhou 510225 China; ^3^ Key Laboratory of Green Processing and Intelligent Manufacturing of Lingnan Specialty Food Ministry of Agriculture Zhongkai University of Agriculture and Engineering Guangzhou 510225 China; ^4^ Academy of Contemporary Agricultural Engineering Innovations Zhongkai University of Agriculture and Engineering Guangzhou 510225 China

**Keywords:** braised squabs, color difference, drip loss rate, physical and chemical properties, shear force

## Abstract

Braised squabs are traditional Chinese foods. However, the processing is highly experience dependent and lacks a theoretical basis. Hence, a comparative study of the physicochemical properties in different processing stages of braised squabs was necessary. We observed the physicochemical changes in the processing stages of braised squabs (raw meat, braised meat, and fried meat). The color parameters, moisture content, and drip loss rate gradually decreased during the processing. On the contrary, crude protein content and pH value were upregulated in the processing stages of braised squabs. Furthermore, the diameter of muscle fiber significantly increased in the braised meat and further decreased in the fried meat compared with the raw muscle fiber. Similarly, hardness, springiness, and chewiness were also increased in the braised step and decreased in the fried step. Additionally, the contents of essential amino acids remain unchanged. Hence, our results provided a certain reference value on the production of braised squabs.

## Graphical abstract

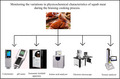

## 1 INTRODUCTION

Braised squab, a famous Cantonese dish, has a crisp taste outside and tender texture inside. The history of squab consumption could trace back to ancient Egypt and subsequently eating squabs became widespread in Europe and Asia (Kokoszynski et al., 2020). Nowadays, China is the largest producer of squab, which has occupied nearly 80% of global production, reaching 680 million squabs. USA, Canada, Great Britain, France, and Italy are also major squab producers, among them American breeders produce about 2.5 million squabs every year (Kokoszynski et al., 2020). Squab meat contains a variety of amino acids, trace elements, and other nutrients. Compared with other poultry, squab has a higher content of protein and lower fat content (Mao et al., 2018; Ye et al., 2018). It has been reported that squab meat was regarded as a potential nutrient supplement (Zhang et al., 2017). For example, squab meat tends to have functionalities, such as preventing aging and hair loss (Hocquette et al., 2010).

The production of pigeon meat is huge worldwide; however, the industrial production of braised squab is relatively backward, which is mostly produced in workshops or restaurants (Kokoszynski et al., 2020). Furthermore, the processing and quality control of braised squab are mainly based on the experience of chef, local consumers’ eating habits, and cultural factors. Although the output of pigeon products was huge, most of them relied on the empirical production and lacked industrialization standards. This lack of scientific basis has severely restricted the industrialization of braised squabs. The quality of braised squabs could not be unified in workshops or restaurants as well. The obvious increase in pigeon products output attracted a lot of attention of scholars. Qian et al. showed the flavor changes of braised squabs (Qian et al., 2021), while this research mainly explored the physical and chemical changes during the processing of braised squabs. Physical and chemical properties of braised squabs included meat color, protein and water content, pH value, drip loss rate, and tenderness, which had a great effect on the sensory quality, processing quality, and nutritional value (Mir et al., 2017; Qamar et al., 2019). Therefore, it is critical to explore the physical and chemical properties of the braised squabs during the processing and to promote their industrialization.

Hence, the purpose of this research was to investigate the physical and chemical properties, fiber microstructure, and amino acid content changes in the processing of the braised squabs (the raw meat step, braising step, and frying step) and provided a certain reference value.

## 2 MATERIALS AND METHODS

### 2.1 Materials and reagents

Squabs (28 days old, male, 250 g) were provided by Guangzhou Restaurant Co., Ltd, Guangzhou, China. Condiments, such as salt, spices, monosodium glutamate, and soy sauce, were purchased from Carrefour Supermarket (Haizhu District, Guangzhou). Sulfuric acid, boric acid, sodium hydroxide, ethanol, ketone sulfate, potassium sulfate, methyl red, bromocresol green, amino acid standard, concentrated hydrochloric acid, phenol, sodium citrate buffer solution, and ninhydrin solution were supplied by Sigma‐Aldrich (St. Louis, MO, USA).

### 2.2 Instruments and equipment

Color difference instrument (NS800) was supplied by Shenzhen SAN 'en Shi Technology Co., Ltd. Fully automatic Kjeldahl Apparatus (K1100) was purchased from Jinan Haineng Instrument Co., Ltd. Texture analyzer (TMS‐Pro FTC) was obtained from Nanjing Mingao International Co., Ltd. Electron microscope (EVO 18) was supplied by Carl Zeiss AG Co., Ltd. pH meter was purchased from Sentron Co., Ltd.

## 3 METHODS

### 3.1 Preparation of braised squabs

Processing technology of braised squabs included visceral removal, rinsing, curing, modelling, braising, and frying.

Squabs (*n* = 30) were culled and their viscera were removed. The squabs were hung up and struck in a water bath for an instant (110 V, 1–2 s), and the feather was removed by suspending in hot water (60–65°C) for 5 min. The whole squab breasts on both sides were removed and regarded as raw meat. Spices, including octagon, pepper, black pepper, cinnamon, orange peel, balsam leaf, fennel, and licorice, were evenly spread to the skin of squabs and preserved for 3 h. The spices were added to water. After boiling, the spices were simmered at 80°C for another 60 min before utilizing (braised soup). Then, squabs were added to the braised soup and simmered at 80°C for 30 min. Samples of braised meat were obtained from this step after cooling. Then, the braised meat was smeared with crisp skin water (1.6% starch, 1.6% honey, and 0.8% vinegar). For the preparation of the fried squab meat, the braised meat was fried in a hot oil pan at 160°C for 3 min, until the core temperature of the squab meat reaching 65°C. This step ended with the samples of fried squab meat. Finally, the fried squabs were cooled by cold air in an artificial climate box at 4°C with an average wind speed of 1 m/s until core temperature was less than 12°C.

After cooling, the squabs were placed in a chilled container with ice bags to maintain a low temperature (0–4°C). Those samples were transported to the laboratory immediately. Then, the breast meat was collected and samples for measuring amino acids were stored in a refrigerator at −80°C and the other samples were stored at 0–4°C until further use.

### 3.2 Color difference analysis and measurement

For the measurement of skin color, the cooked braised squabs were prepared as previously described by Wen et al. (2020). Briefly, Color parameters L*, a*, and b* allowed to identify, count, and measure objective variances between the different colors. The braised squabs were naturally cooled to room temperature, and the samples (squab breast) were cut into 3 × 3 × 1 cm square slices, and the color characteristic values such as *L* ^*^ (lightness), *a* ^*^ (redness), and *b* ^*^ (yellowness) were measured and evaluated by spectrophotometer. The standard white plate was used to calibrate the colorimeter. This difference, consisting of deviations Δ*L* ^*^, Δ*a* ^*^, and Δ*b* ^*^, was best expressed by the term ΔE, which was a square root of the sum of the individual deviation squared. The individual differences ΔE in *L* ^*^, *a* ^*^, and *b* ^*^ values were calculated from the formula Δ*E* = [(Δ*L* ^*^)^2^ + (Δ*a* ^*^)^2^ + (Δ*b* ^*^)^2^]^1/2^ (Wen et al., 2020).

### 3.3 Determination of crude protein and moisture contents

The crude protein content of muscle samples was analyzed by the Kjeldahl procedure (AOAC method number 920.39) (Matis et al., 2019). The moisture content was determined by the gravimetric method as described in AOAC (Gaithersburg, 2007) and the analytical standards of the Instituto Adolfo Lutz (Blank et al., 2017).

### 3.4 Determination of pH value

The pH values of the raw, braised, and fried meat were determined with a Sentron pH meter according to the method of Fletcher et al. (2000). Before determination, the pH meter was calibrated with the standard solution. Briefly, approximately 5 g of meat tissue was removed from the posterior portion of the breast. The pH meter electrode was inserted into the incision (1 cm) of squab breast meat to read the pH values.

### 3.5 Determination of the rate of drip loss

The water‐holding capacity of the meat was estimated by measuring the drip loss of the different samples. The rate of drip loss was measured using the method of Honikel (1998) with slight modifications. The squab breast meat was cut and weighed (W1, 1 × 1 × 3 cm). A wire (20 cm) was shaped to form "M", and hung in the paper cup to prevent the meat from touching the inner wall of the paper cup. Then, the paper cup was sealed with a sealing pocket and placed in the refrigerator at 4°C for 24 h. The wire was removed and samples were weighed accurately (W2). The water loss was calculated according to the following formula:

$$W=\frac{W_{1}‐W_{2}}{W_{1}}\times 100\%$$

Where *W*_1_ and *W*_2_ are the weight of samples before and after 24 h, respectively, and W is the rate of drip loss.

### 3.6 Determination of muscle fibers and microscope observation

The distribution characteristics of muscle fibers were determined as described by Cheng et al. (2020) and Palka and Daun (1999) with minor modification. Small pieces (1 × 1 × 1 cm) from squab breast meat at different stages were fixed in 2.5% glutaraldehyde solution at 4°C for 1 day and rinsed with 0.1 mol/L phosphate buffer solution (pH 7.4) for three times. Then, samples were dehydrated by gradient ethanol solutions of 30%, 50%, 70%, 80%, and 90%, respectively for 10 min. Moreover, 100% ethanol was used to dehydrate these samples for another 15 min. These dehydrated steps were repeated three times. The dehydrated samples were frozen in liquid nitrogen and freeze‐dried until further use. The fragments of dried tissue were fixed on holders with silver cement. Then, the specimens were placed and photographed with a 10 kV acceleration voltage and a working distance of 10 ± 13 mm to observe the microstructure of squab meat under an electron microscope.

### 3.7 Determination of shear force

The shear force was determined using the method of Wattanachant et al. (2005). The squab breast meat was cut in the direction perpendicular to the muscle fiber (3 × 3 × 1 cm) for shear analysis using the Texture Analyzer equipped with a Warner–Bratzler shear apparatus. The operating parameters consisted of a crosshead speed of 2 mm/s. Each core was sheared with a perpendicular fiber orientation once, and the peak of the shear force profile was regarded as the shear force value. The average shear force of the five cores from each experimental unit was reported.

### 3.8 Determination of texture profile analysis

The texture profile analysis (TPA) parameters, including hardness, adhesiveness, cohesiveness, springiness, gumminess, and chewiness, were measured using the method of Herrero et al. (2008). The samples (squab breast meat, 1 × 1 × 1 cm) were cut in the direction perpendicular to the muscle fibers. The texture measurements were performed using a texture analyzer (TA. XTPlus, Texture Technologies, Hamilton, MA) with a cylinder probe to compress the samples. A 50‐kg load cell at a test speed of 3.0 mms‐1 (pre‐test), 1.0 mms‐1 (test), and 3.0 mms‐1 (post‐test) was used to reach a 50% compression. The parameters, hardness (maximum force required to compress the sample), springiness (the ability of the sample to recover its original form after deforming force), adhesiveness (area under the abscissa after the first compression), and cohesiveness (the extent to which the sample could be deformed prior to rupture) were calculated (Herrero et al., 2008).

### 3.9 Determination of amino acids

The levels of amino acids in the samples of squab breast were analyzed by an amino acid analyzer (L‐8900, Hitachi, Japan) as described by Li et al. (2017) with slight modifications. Absorbance was recorded at 570 nm and 440 nm. The injection volume was 20 μL. The relatively quantitative analysis of the amino acids was calibrated by the external standard method. The standard mixture solution (the concentration of Cys‐Cys was 1.25 μmol/ml and other amino acids were 2.5 μmol/ml) was diluted to make standard curves.

#### 3.9.1 Statistical analysis

Data were shown as the mean ± SE from the experiments. Statistical analysis was performed using Tukey–Kramer multiple comparison tests as indicated in each table and figure legend. The statistical significance level was set at *p* < 0.05 using SPSS17.0.

## 4 RESULTS AND DISCUSSION

### 4.1 The color parameters

The color of the products is one of the important characteristics that largely contributes to consumption tendency. The color parameters (L*, a*, and b*) and color difference (Delta E) of squab before and after the cooking process are shown in Table 1. The cooking process of braising significantly (*p* < .05) decreased the L* values of squab skin, from 60.04 to 23.43, compared with the fresh meat. After frying, the L* values decreased significantly. The fresh breast meat had the a* value of 7.35 which was significantly higher than the braised squab meat (5.38), but lower than that of fried squab meat (13.59). The b* values of squab meat facilitated inverse changes compared to the a* values in the whole cooking process, whilst the b* values increased from 18.87 to 24.15 after braising and then decreased to 13.96 after the frying process.

**TABLE 1:** The changes in color value and color difference (Delta E) of braised pigeon during processing

	Raw meat	Braised meat	Fried meat
L*	60.04 ± 0.08380^a^	43.9 ± 0.02867^b^	23.43 ± 0.03559^c^
a*	7.35 ± 0.05354^b^	5.38 ± 0.00943^c^	13.59 ± 0.02055^a^
b*	18.87 ± 0.04190^b^	24.15 ± 0.06236^a^	13.96 ± 0.01886^c^
Δ*E*	63.36 ± 0.06284^a^	50.39 ± 0.02459^b^	30.47 ± 0.03028^c^

Note

The levels of color value changes of braised squabs were measured. Values were shown as the mean ± SE (*n* = 5). Value without a common letter in each row differs significantly among groups (*p* < .05) by Tukey–Kramer multiple comparison test.

The changed color values of squab meat after braising might be due to the color pigments of spices (Qamar et al., 2019). On the other hand, the decrease of a* value might associate with the oxidation of ferrous myoglobin to ferric myoglobin in the braising stage (Bak et al., 2019; Cheftel & Culioli, 1997). The increase of a* values after frying might be related to the high‐temperature heating process. Similar results were reported by Ortuno et al. (2021), it has been reported that protein denaturation that occurred above 60°C reduced the typical red color of meat and increased surface light reflectance. Furthermore, the type of the honey also affected the color of the pigeon meat. The effects of other types of honey on the color of pigeon meat will be further determined in subsequent experiments.

### 4.2 The change of moisture content

**FIGURE 1:**
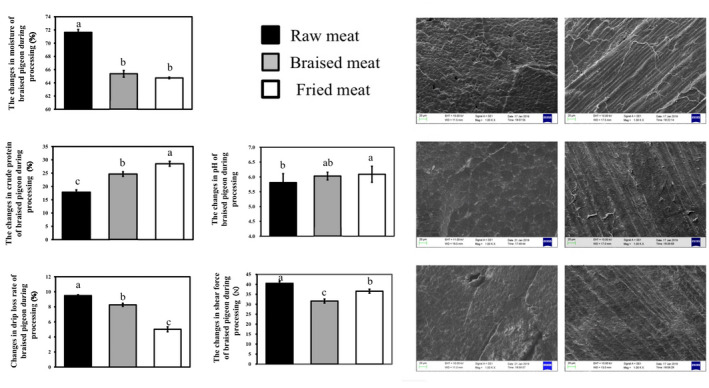
The changes in moisture (A), crude protein (B), pH (C), drip loss rate (D), and shear force (E) of braised squabs during processing. The levels of moisture (A), crude protein (B), pH (C), drip loss rate (D), and shear force (E) changes of braised squabs were measured. Furthermore, the levels of (a) transversal section of raw meat step, (b) longitudinal section of raw meat step, (c) transversal section of braised step, (d) longitudinal section of braised step, (e) transversal section of fried step, and (f) longitudinal section of fried step changes are shown and measured in the Figure 1(f). Values were shown as the mean ± SE (*n* = 5). Different superscripted letters indicate significant differences between the groups by Tukey–Kramer multiple comparison test (*p* < .05).

The braising and frying processes of squab meat significantly affected the moisture content of the meat. Compared with raw meat, the moisture in braised meat and fried meat were significantly decreased (Figure 1a). The moisture content of braised meat was decreased from 71.64% in raw meat to 65.37%. Furthermore, after high‐temperature frying, the water content further decreased by 0.76% (Figure 1a).

It has been reported that a long heating time during braising process denatures myofibril, resulting in a shrinkage of meat product and myofibril accumulation. The process decreased water retention in muscles (Lagerstedt et al., 2008). Water was probably lost from the samples owing to heat‐induced protein denaturation during cooking, which caused less water to be entrapped within the protein structures. Furthermore, after high‐temperature frying, the moisture content was slightly decreased with possible speculation that surface moisture was emitted during short frying time (3 min) (Kim et al., 2015).

### 4.3 Change of protein content

During the cooking process, we examined the modifications in crude protein contents of the breast meat. As shown in Figure 1b, the content of crude protein of breast meat was significantly raised in the braising and frying processes (*p* < .05). A similar result was reported in cooking beef, which was stated that the crude protein content was gradually increased (Kim et al., 2015). The increase in protein content could be explained by the reduction in moisture (Kim et al., 2015). Another explanation might involve nonprotein nitrogen. It has been reported that increasing the content of nonprotein nitrogen might be import errors in this complicated condition. Zhang et al. (2013) detected a similar result that the composition of nonprotein content in crucian carp soup was increased with the increase in cooking temperature.

### 4.4 The change in pH during the processing of braised squabs

The change in pH during the processing is shown in Figure 1(c). This result revealed that the pH value was significantly increased in fried meat, and reached 6.09 after processing. Moreover, the flavoring substance of glutamate sodium, an important umami substance in meat products, could affect the pH value of the meat (Dwivedi & Arnold, 1973; Zhang et al., 2012). The pH values of the braised and fried meat were higher than that of raw meat, which was probably caused by the heating process. The loss of lactic acid caused a rise in pH value (Wattanachant et al., 2005); Another explanation was that the chemical bonds of protein structure were broken, which reduced the acid group of muscle protein, according to the report of Ma and Ledward (2004). They detected that the increase in pH value was related to temperature. High temperature could destroy the chemical bond energy of stable protein structure (such as hydrophobic interaction, hydrogen bond). Last, but not least, the addition of spices, including their proportions, also could influence the pH value, similar results were reported by Ayofemi (2018).

### 4.5 The change in drip loss rate during the processing of braised squabs

It has been confirmed that the water loss rate could objectively reflect the water retention of squab meat. The drip loss rate among raw, braised, and fried meat was obviously decreased by the heating process. Compared with the raw meat, the drip loss rate of braised meat was significantly decreased from 9.36% to 8.01% (Figure 1d). In addition, after the frying process, the drip loss rate was further decreased to 4.77%. It was reported that the drip loss rate was decreased with the increase of pH value (Holmer et al., 2008). Similar results of drip loss rate and pH value were detected in this report.

### 4.6 The change in shear force during the processing of braised squabs

As shown in Figure 1e, the change in the shear force of squab meat during the cooking process was examined. Braised meat contained the lowest shear force (31.62N), compared with raw meat and fried meat, while the shear force rose to 36.54 N after the frying process.

The possible reason was caused by the thermal change of collagen (Lagerstedt et al., 2008). With the temperature increase, the collagen of squab meat was degraded and formed gelatin. These changes decreased the shear force in braised meat (Ishioroshi & Jima, 1979; Liu & Foegeding, 1996; Qiao et al., 2002). These results matched the report of Vasanthi et al. (2007), which found that the collagen solubility increased with temperature and time of cooking in the water baths. The subsequent increase of shear force might be mainly due to the structural changes and dissolution of gelatin, leading to the decrease of muscle fiber integrity (Fritz & Dietrich, 1992; Ishioroshi & Jima, 1979; Liu & Foegeding, 1996; Locker, 1984; Qiao et al., 2002). The increasing shear force in the fried step had a direct relation with the contraction of muscle fibers and the destroyed structural integrity of fascia and endomysium, which was in an agreement with our data of muscle fiber structure. The data illustrated that the diameter of the transversal section of the muscle fiber structure was decreased in the frying process (Figure 1f) and Vasanthi et al. (2007) found that marked discontinuity and distortion of both endomysium and perimysium with loss of structural integrity were observed in meat cooked under high temperature. Hence, these results clarified that braising process‐induced protein denaturation and gelatin formation decreased the shear force. The increasing shear force of squab meat after the fried step was caused by muscle fibers contraction and the destroyed structural integrity.

### 4.7 The change in muscle fiber structure during the processing of braised squabs

The microstructure of squab meat after cooking is shown in Figure 1F. Raw muscle fibers were relatively intact and irregular in shape (Figure 1F(a)) and there were some spaces between the muscle fibers (Figure 1F(b)). In the braised stage, the diameter of the muscle fiber was significantly increased and the muscle fibers presented relatively regular polygons (Figure 1F(c and d)). The frying process decreased the diameter of muscle fiber with relatively low integrity (Figure 1F(e and f)).

The mechanism of increasing muscle fiber diameter was that the perimysium membrane tightly wrapped the muscle fibers in the raw meat and the endomysium and perimysium were separated from the muscle fibers after heating. The muscle fibers gradually elongated and expanded in the horizontal and longitudinal directions, which was the reason for increasing the diameter and decreasing the space of muscle fibers. These results matched the research of Palka and Daun (1999).

In addition, the frying process decreased the diameter and integrity of muscle fiber (Chan et al., 2015). The shrinkage might be due to the thermal denaturation of myosin and actomyosin complex and a large decrease in water retention. Offer et al. also reported the changes of meat shrinkage during cooking. Firstly, the myosin and the proteins that constitute the myofibrillar membrane were denatured by heating. Thus, the structure of the myofibrillar membrane was destroyed. Then, the binding force of myofibrils to water and the structural integrity of myofibrils were reduced. Last, the contraction of the myofibrils was increased (Offer et al., 1989; Palka & Daun, 1999).

### 4.8 The change in TPA parameters during the processing of braised squabs

TPA is a mechanical method to objectively evaluate the quality of products by simulating oral chewing. As shown in Table 2, the braised squabs showed a bell‐shaped increase in hardness, springiness, and chewiness. Adhesiveness, cohesiveness, and gumminess revealed a decreasing tendency. The hardness was increased (5.88%) after braising and then decreased after frying. The value of adhesiveness was decreased to 0.1324 (79.24%) by braising and increased to 0.1873 (41.47%) by frying compared to the raw meat. In addition, there was no significant change in the cohesiveness between raw and cooked meat, though fried meat only showed a reduction tendency from 0.6 to 0.5. Next, compared with braised meat, the frying process decreased springiness, gumminess, and chewiness (7.3%, 14.91%, and 22.22%, respectively).

Hardness, springiness, and chewiness were negatively related to shear force, which was in agreement with the results of U‐Chupaj et al. (2017). It has been reported that these changes were closely related to temperature, protein structure, and cooking time (Cheng et al., 2020; Palka & Daun, 1999; Ripoll et al., 2019). Springiness, chewiness, and hardness were matched with Palka and Daun (1999), who reported that the thermal conditions had different effects on the TPA parameters and parameters had a close relation with the diameter of the fiber and the degree of fiber swelling. The maxima of TPA parameters (springiness, chewiness, and hardness) were observed in the range 70–100℃ by Palka and Daun (1999). Springiness reached a maximum at 70℃ and chewiness showed a maximum at 80℃, whereas maximum hardness occurred in the range 80–100℃. Hence, these values revealed a bell‐shaped increase and reached the maximum value in braised step. In the area of the mechanism of decreasing parameters, the possible explanation was that the heating process not only contracted myofibrils and broke hydrogen bond and hydrophobic bond, but also destroyed the structural protein systems of meat, as actomyosin complex and collagen, and caused moisture content decrease. Up to 70℃, myosin, actin, and collagen were denaturized by heating (Palka & Daun, 1999). Hence, when temperatures higher than 80℃, the tenderizing effect of meat was probably caused by the gelatinization of meat collagen. Another possible reason was that the TPA parameter changes also depended on the cooking time. Machlik and Draudt (1963) reported that heating meat at 71℃ decreased the toughness of beef during the first 9 min of cooking. Therefore, discrepancies between temperature‐induced TPA parameter changes and cooking time–increased TPA parameter changes in this research need to be clarified in the future (Table 2).

**TABLE 2:** The changes in TPA of braised squabs during processing

Sample	Raw meat	Braised meat	Fried meat
Hardness (*N*)	56.84	60.18	49.77
Adhesiveness (*N*)	0.6377	0.1324	0.1873
Cohesiveness (Ratio)	0.6	0.6	0.5
Springiness (mm)	2.81	2.87	2.66
Gumminess (*N*)	32.08	31.72	26.99
Chewiness (mJ)	90.7	92.7	72.1

### 4.9 The change in amino acid content during the processing of braised squabs

Amino acids play important roles in the flavor formation of braised squabs. Amino acids act as flavor precursors or react directly to produce aroma substances (Yvon et al., 1997). The flavor characteristics of various flavor substances not only depended on the variety of amino acids but also relied on their threshold values (Dang et al., 2008). Subsequently, we examined the changes in amino acid content during the processing. Amino acid contents in the processing of braised squabs are shown in Table 3. During the process, the total content of various flavor amino acids remained unchanged. The relative content of bitter amino acid was the highest. The total content of amino acid in raw meat was 17.65 g/100 g. Umami amino acids, sweet amino acids, and bitter amino acids accounted for 24.54%, 21.41%, and 42.66%, respectively. Similar results were observed in the braised and fried samples. The total content of amino acids of braised meat and fried meat were 24.16 g/100 g and 24.25 g/100 g, respectively. Glutamate had the highest threshold. Hence, it had directly related to the flavor formation of braised squabs. Moreover, although the content of bitter amino acids was relatively high, sodium chloride could inhibit its bitter taste through the interaction with bitter amino acids (Keast & Breslin, 2002). Therefore, the flavor of braised squabs firstly came from the direct contribution of glutamate. On the other hand, it was the result of the interaction of amino acids. Moreover, it has been reported that the increase in amino acid content in the braised meat was the result of the hydrolysis of squab meat protein during the heating process (Scannell et al., 2004). Similarly, the increasing content of the amino acid was detected in this research from 17.65 g/100 g to 24.16 g/100 g.

**TABLE 3:** The changes in amino acids during processing of braised squabs

Amino acids	Raw meat (g/100 g)	Braised meat (g/100 g)	Fried meat (g/100 g)	The threshold value (g/100 ml)
Glutamate (Glu)	2.65	3.67	3.68	0.03
Aspartic acid (Asp)	1.7	2.3	2.31	0.1
∑UAA	4.35	5.97	5.99	N.D.
Alanine (Ala)	1.32	1.72	1.76	0.06
Glycine (Gly)	0.84	1.15	1.2	0.13
Serine (Ser)	0.75	1.03	1.04	0.15
Threonine (Thr)^a^	0.87	1.19	1.2	0.26
∑SAA	3.78	5.09	5.2	N.D.
Histidine (His)	0.59	0.76	0.76	0.02
Isoleucine (Ile)^a^	0.9	1.25	1.24	0.09
Valine (Val)^a^	0.97	1.34	1.35	0.04
Phenylalanine (Phe)^a^	0.88	1.2	1.2	0.09
Methionine (Met)^a^	0.52	0.74	0.75	0.03
Arginine (Arg)	1.29	1.78	1.77	0.05
Tyrosine (Tyr)	0.74	1.03	1.01	N.D.
Leucine (Leu)^a^	1.64	2.25	2.25	0.19
∑BAA	7.53	10.35	10.33	N.D.
Proline (Pro)	0.45	0.66	0.67	0.3
Lysine (Lys)^a^	1.54	2.09	2.06	0.05
Total amino acid content	17.65	24.16	24.25	N.D.

^a^ Represents essential amino acid, UAA, Umami amino acids; SAA, sweet amino acids; BAA, Bitter amino acids; Abbreviations see Table 2. The amounts in amino acids during processing of braised squabs were based on the wet weight.

## 5 CONCLUSIONS

In conclusion, our findings highlight the physicochemical properties of braised squabs during the processing. Firstly, the color difference among these steps was gradually decreased. Compared with raw meat, the moisture content and drip loss rate of the fried meat were significantly decreased. On the contrary, crude protein content and pH value were upregulated by the processing. Furthermore, the heating process decreased the structural integrity of myofibrils. Raw muscle fibers were loosely arranged and there were some spaces between the muscle fibers. In the braised and fried process, muscle fiber diameter showed a bell‐shaped increase and presented relatively regular polygons. The space between fibers in the braised process gradually decreased. Moreover, hardness, springiness, and chewiness, some important attributes in texture profile analysis, showed bell‐shaped increases and a negative correlation with shear force. Adhesiveness, cohesiveness, and gumminess revealed decreased trends. Finally, the total content of various flavor amino acids remained unchanged during the process. Through the research, the quality of braised squab would change significantly during processing. Processing temperature and processing time played crucial roles in the industrialization of braised pigeons, which would affect the color, pH, water retention, and muscle fiber morphology of pigeon meat. Therefore, processing temperature and processing time should be considered in the industrialization process of braised pigeons. Our findings provided some theoretical basis for physicochemical properties of braised squabs during processing and laid a foundation for the quality control and process control of industrial production.

## CONFLICTS OF INTEREST

None of the authors has any financial or other interest that could inappropriately influence or bias the content of this manuscript.

## AUTHOR CONTRIBUTION

**Wenhong Zhao:** Formal analysis (equal); Funding acquisition (equal); Investigation (lead); Writing – original draft (supporting). **Qiaoyu Liu:** Conceptualization (lead); Resources (equal). **Hao Jiang:** Formal analysis (lead); Investigation (lead); Project administration (lead); Supervision (lead). **Minyi Zheng:** Data curation (equal); Resources (equal); Software (equal); Writing – original draft (equal). **Min Qian:** Data curation (equal); Formal analysis (equal); Software (equal); Validation (equal). **Xiaofang Zeng:** Data curation (lead); Funding acquisition (equal); Visualization (equal). **Weidong Bai:** Funding acquisition (lead); Supervision (supporting).

## ETHICAL APPROVAL

The housing and treatment of the animals were carried out following the national and international laws as well as with the institutional guidelines. All animal/meat used in this study were obtained from Guangzhou Restaurant Co., Ltd, Guangzhou, China. We were explicitly issued a formal waiver of ethics approval.

## Data Availability

The data that support the findings of this study are available from the corresponding author upon reasonable request.
